# On center field or at the sidelines? – A plea for a multimodal approach between medical schools and medical student communities while integrating international medical students

**DOI:** 10.3205/zma001207

**Published:** 2018-11-30

**Authors:** Timo Astfalk, Danmei Zhang, Ricardo Patricio Pérez Anderson, Obada T. Alhalabi, Henrike Schulze

**Affiliations:** 1 University Medical Center Rostock, Rostock, Germany; 2 Project International medical students at the German Medical Students' Association (bvmd), Berlin, Germany; 3 Ludwig-Maximilians-University Munich, Munich, Germany; 4 University of Heidelberg, Heidelberg, Germany; 5 Medizinische Hochschule Hannover, Hannover, Germany

**Keywords:** International medical students, cultural diversity, social learning, integration, curriculum development

## Abstract

An increasing number of medical schools offer support services for international students. Their frequent focus on linguistic and academic support often reflects a rather deficit-oriented perception of international medical students. Therefore, this comment advocates a stronger focus on the integration into the local medical student communities. This happens both against the background of the expected academic benefits and the added value of intercultural learning at medical schools.

## Zusammenfassung

Eine steigende Zahl medizinischer Fakultäten bietet Unterstützungsangebote für ihre internationalen Studierenden an. Solche Angebote fokussieren häufig auf die sprachliche und fachliche Unterstützung von internationalen Medizinstudierenden und spiegeln damit eine eher defizitorientierte Wahrnehmung dieser Studierendengruppe wider. Dieser Kommentar plädiert daher für eine verstärkte ergänzende Integrationsförderung in die lokalen Studierendenschaften. Dies geschieht sowohl vor dem Hintergrund des zu erwartenden akademischen Nutzens als auch des Mehrwerts interkulturellen Lernens an medizinischen Fakultäten.

## Introduction

Reports of lower academic performance [1], [2], [3] as well as university-related issues [4], [5] by international medical students have led to the establishment of several support programs in recent years [6], [7], [8], [9], [10], [11]. Oftentimes, these programs foreground the aspects of linguistic [10], [11] and academic [9] support. Despite these initiatives, however, there remains a perception among faculty members and study deaneries that the academic issues of international medical students have not yet improved [6], [12]. In addition, focusing on performance differences reinforces a deficit orientation towards international students [13]. We therefore propose to place a greater emphasis on the aspect of integrating international students into the local medical student communities. This offers the potential to make greater use of the benefits of the social learning environment [14], [15] and cultural diversity [16] in medical student communities. We would also like to show why the early inclusion of a student perspective in the process of planning and implementing support programs for international medical students is beneficial to this goal. 

## Integration and academic success in medical school

Ever since Tinto's model on student dropout, the academic and social integration at universities as well as individual characteristics are considered relevant for academic success [17]. Although empirically difficult to grasp, this correlation has been repeatedly confirmed by past research [14], [15], [18]. While looking at the study of medicine from the students' perspective, there are several explanations for this connection. On one side, the medical student community can provide information for the organization of one's academic courses or during exam preparation. On the other side, personal learning is also shaped by the environment in a way that subject-related discussions in learning groups can accurately model future oral examination situations (for students). Last but not least, from day one the embeddedness in the medical student community leads to a strong sense of connectedness and support that is rarely found in other fields of study. This bond among medical students is also considered to be the foundation of a shared student identity, which later evolves into a professional identity [19].

## Difficulties with the integration of international medical students

Previous research [20], [21] described a generally easy integration of new medical students in the medical student community. Nevertheless, international medical students seem to experience much more difficulties during this process. Medical students with a migration background report increased loneliness during the semester [22], fewer contact to German students and lower perceived support by them [5], [23]. As a consequence, social network analyses found cultural and linguistic boundaries in medical school classes that suggest a certain degree of segregation of linguistic and cultural circles [24], [25]. It can be assumed that both international and domestic students would benefit to a high degree from a better integration of international students. In addition to the advantages described above, additional positive effects on the language competences of international students can be expected [16]. Furthermore, intercultural learning among domestic and international students is often only made possible through a strong interaction between these groups [16].

## Supporting the integration of international medical students

Against this background, support programs for international medical students should be reviewed and adapted in ways that promote further integration. Multimodal programs play a central role here as they influence various factors that are important for integration processes. While language and academic tutorials are well-established means to develop the learning competences of international students, communicative and intercultural trainings are better to teach skills that aid the interaction with the local medical student community [26]. Also, the effects of established and culturally-influenced structures in medical schools as well as the medical student community must be considered. Therefore it makes sense to evaluate the cultural openness of existing student and academic events. Another facet of promoting integration is by increasing the visibility of cultures at higher education institutions, which includes the establishment of meeting places and the empowernment of international students. The inclusion of local student communities in the conception and implementation of such offers is essential and can be witnessed in projects that have been successful for many years [7], [8], [9]. This approach supplements official support programs of faculties by promoting a stronger connection within the respective classes at medical schools and also enables an easy approach to the target group of international students. Through such cooperations, support programs can create offers that are based on actual needs, increase their efficiency and reduce the deficit orientation towards international students. If this cooperation between the medical student community and the faculty is successful, sustainable academic and social integration can be achieved and the potential of internationalization [27] at medical schools can be used more optimally. As a result, medical schools can help safeguard the quality of medical education while contributing to current debates on migration policies.

## Competing interests

The authors declare that they have no competing interests. 
